# Control of synaptic transmission and neuronal excitability in the parabrachial nucleus

**DOI:** 10.1016/j.ynpai.2020.100057

**Published:** 2020-12-14

**Authors:** Nathan Cramer, Gleice Silva-Cardoso, Radi Masri, Asaf Keller

**Affiliations:** a Department of Anatomy and Neurobiology and the Program in Neuroscience, University of Maryland School of Medicine, Baltimore, MD 21201, USA; b Department of Oral Sciences and Pain. School of Dentistry, University of Maryland Baltimore, Baltimore, MD, USA; c Department of Psychology, Faculty of Philosophy, Sciences and Letters of Ribeirão Preto, University of São Paulo, Brazil

**Keywords:** Parabrachial nucleus, Inhibition, Excitation, Synaptic modulation

## Abstract

The parabrachial nucleus (PB) is a hub for aversive behaviors, including those related to pain. We have shown that the expression of chronic pain is causally related to amplified activity of PB neurons, and to changes in synaptic inhibition of these neurons. These findings indicate that regulation of synaptic activity in PB may modulate pain perception and be involved in the pathophysiology of chronic pain. Here, we identify the roles in PB of signaling pathways that modulate synaptic functions. In pharmacologically isolated lateral PB neurons in acute mouse slices we find that baclofen, a GABA_B_ receptor agonist, suppresses the frequency of miniature inhibitory and excitatory postsynaptic currents (mIPSCs and mEPSC). Activation of µ-opioid peptide receptors with DAMGO had similar suppressive effects on excitatory and inhibitory synapses, while the κ-opioid peptide receptor agonist U-69593 suppressed mIPSC release but had no consistent effects on mEPSCs. Activation of cannabinoid type 1 receptors with WIN 55,212-2 reduced the frequency of both inhibitory and excitatory synaptic events, while the CB1 receptor inverse agonist AM251 had opposite effects on mIPSC and mEPSC frequencies. AM251 increased the frequency of inhibitory events but led to a reduction in excitatory events through a GABA_B_ mediated mechanism. Although none of the treatments produced a consistent effect on mIPSC or mEPSC amplitudes, baclofen and DAMGO both reliably activated a postsynaptic conductance. These results demonstrate that multiple signaling pathways can alter synaptic transmission and neuronal excitability in PB and provide a basis for investigating the contributions of these systems to the development and maintenance of chronic pain.

## Highlights

• The parabrachial nucleus (PB) processes intero- and exteroceptive noxious stimuli.
• Synaptic activity in PB is regulated by GABA_B_, µ- and κ-opioid and CB1 receptors.
• GABAergic presynaptic terminals are most potently regulated by these receptors.
• Changes in these pathways may promote PB excitability and pathological conditions.

## 1 Introduction

The parabrachial nucleus (PB) subserves sensory, homeostatic and aversive functions (Palmiter, 2018, Chiang et al., 2019, Chiang et al., 2020), and is a critical hub for nociception (Gauriau and Bernard, 2002, Han et al., 2015). The role of PB in pain processing is highlighted by its reciprocal connections with brain regions associated with both the sensory and affective aspects of pain (Fulwiler and Saper, 1984, Jasmin et al., 1997, Chen et al., 2017) as well as with regions of descending modulatory control of nociception (Roeder et al., 2016, Chen et al., 2017, Chen and Heinricher, 2019).

In addition to normal nociception, PB also contributes to pathological pain conditions. Using a combination of rat and mouse models, we have shown that chronic pain is causally related to amplification of PB responses, and to reduced inhibition of PB neurons by the central amygdala (Uddin et al., 2018, Raver et al., 2020). These findings suggest that mechanisms that regulate the efficacy of synaptic transmission within PB, as well as the intrinsic excitability of PB neurons, significantly contribute to normal and dysregulated nociception. How this regulation occurs, however, has not yet been determined.

Several neurotransmitter receptors commonly associated with modulation of synaptic transmission are expressed in PB, including those activated by GABA_B_, µ- and κ- opioid (MOP/KOP), and cannabinoid type-1 (CB1) receptors. While the presence of these signaling pathways is known, their functional impact on PB neurons is not. In particular, little is known about how these receptors are distributed between inhibitory and excitatory afferents or whether they exert greater regulation pre- or postsynaptically. Our ability to understand how PB contributes to chronic pain requires understanding how these neuromodulators affect synaptic release and neuronal excitability under normal conditions. This is the goal of this study.

## 2 Results

### 2.1 GABA_B_ receptor activation inhibits synaptic release

**Fig. 1:**
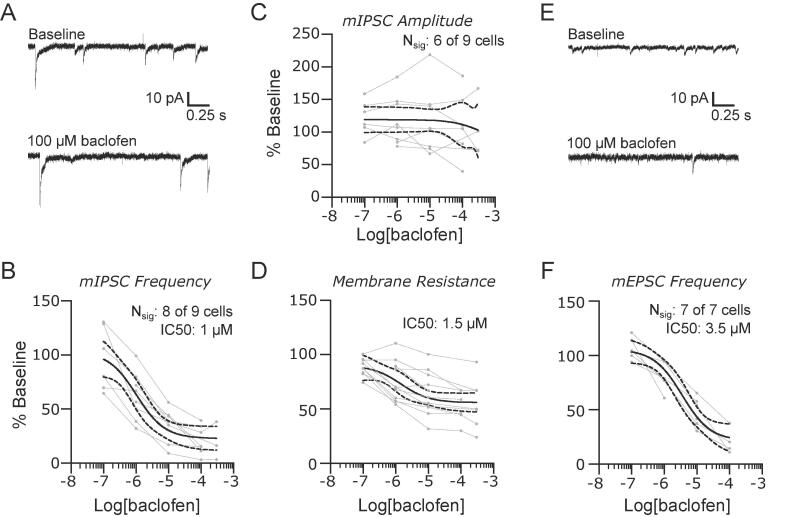
GABAB receptors suppress synaptic input and lower membrane resistance in the lateral parabrachial nucleus. (A) Representative recordings of mIPSCs in baseline conditions and in the presence of 100 μM baclofen. The suppression of mIPSC frequency was dose dependent (B). The amplitudes of mIPSC were not affected (C) despite a dose-dependent reduction in membrane resistance (D). Baclofen also reduced the frequency of mEPSCs. Representative recordings are shown in (E) with group data for mEPSC frequency in (F). Group data are fit with a log(dose) vs response curve ± 95% CIs.

We have previously shown that GABAergic inputs to the PB regulate the expression of pain behaviors. Here, we investigated the effect of GABA_B_ receptor activation by recording pharmacologically isolated mIPSCs and mEPSCs before and after bath application of increasing concentrations of baclofen (0.1 to 300 µM). As shown in the representative mIPSC recordings in Fig. 1A, 100 µM baclofen reduced the frequency of synaptic events. We obtained a dose-response profile by normalizing the median mIPSC frequency at each concentration of baclofen to the baseline value for each neuron. Only neurons that had a significant response to the agonist —determined by Kruskal-Wallis test with p < 0.05—were included (mIPSCs: n = 8 out of 9, mEPSCs: n = 5 out of 5 neurons).The large variability in instantaneous event frequencies (CV: 270 ± 80%, n = 11) and amplitudes (CV: 60 ± 20%, n = 11) in each neuron increases variability in the normalized dose response group data. Despite the inherent variability of these parameters, this analysis (Fig. 1B) revealed an IC50 of 1 µM (95% CI: 0.3 to 4 µM) and a maximum inhibition to 23% (95% CI: 11 to 34%) of baseline activity.

The amplitudes of mIPSCs were significantly altered by baclofen in 6 out of 9 neurons (defined as above for each cell with a Krusal-Wallis (K-W) test with p < 0.05), but the direction of change was not consistent across neurons. For example, mIPSC amplitudes in some neurons decreased uniformly with increasing concentrations of baclofen. In other neurons, small amplitude mIPSCs were suppressed by baclofen while large amplitude events remained unchanged. In these latter cells, the loss of small amplitude events resulted in an increase in the median amplitude in baclofen. As a result, when examined as a population, there was no consistent effect of baclofen on mIPSC amplitude (Fig. 1C), despite a dose dependent decrease in membrane resistance (Fig. 1D; IC50 = 1.5 µM; 95% CI: 0.9 to 23 µM), with a maximum decrease to 56% (95% CI: 44 to 65%) at the highest baclofen concentration of 300 µM.

Baclofen inhibited the frequency of mEPSCs in all 7 neurons recorded (Fig. 1E). Analysis of normalized mEPSC frequency group data (Fig. 1F) revealed a significant effect of baclofen with an IC50 of 3.5 µM (95% CI: 1 to 10 µM) and maximum suppression to 22% (95% CI: 3 to 36%) of baseline. We did not analyze mEPSC amplitude in these recordings because the cesium-based pipette solution blocks potassium channels and alters the membrane resistance.

Together, these data demonstrate that GABA_B_ receptors have both pre- and postsynaptic effects in PB by inhibiting GABAergic and glutamatergic transmission and activating a postsynaptic conductance.

### 2.2 µ and k opioid receptors differentially affect mEPSCs and mIPSCs

**Fig. 2:**
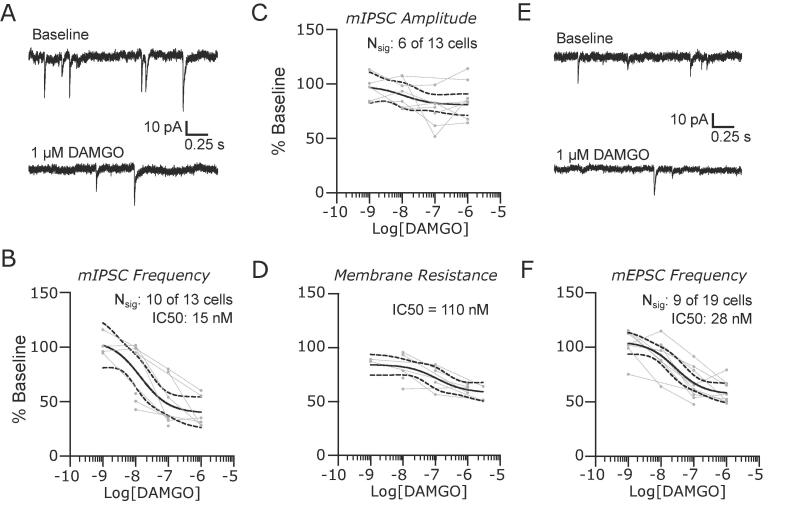
MOP receptors suppress synaptic activity and reduce input resistance in lateral parabrachial neurons. (A) Representative recordings of mIPSCs in baseline conditions and in the presence of 1 µM DAMGO. The suppression of mIPSC frequency was dose dependent (B), but the amplitudes (C) were not affected. However, DAMGO caused a significant dose dependent reduction in membrane resistance in PB neurons (D). Similar results were observed for mEPSCs with representative recordings in (D) and group data for frequency in (F). Group data are fit with a log(dose) vs response curve ± 95% CIs.

Mu opioid peptide (MOP) receptors are highly expressed in PB, suggesting that endogenous and exogenous agonists may also regulate synaptic activity in this nociceptive hub. We tested this prediction directly by recording mIPSCs and mEPSCs in the presence of increasing concentrations of the selective MOP agonist, DAMGO (1 nM to 1 µM). Representative mIPSC recordings in Fig. 2A demonstrate a suppression of synaptic release probability by MOP receptor activation. Normalization of the median mIPSC frequencies following DAMGO application to baseline values revealed a significant dose-dependent effect with an IC50 of 15 nM (95% CIs: 3 to 95 nM, 10 out of 13 neurons responding) and maximum inhibition to 46% of baseline values (95% CIs: 34 to 56%, Fig. 2B). There was no effect of DAMGO on mIPSC amplitudes when examined as a population (Fig. 2C), despite a significant effect in 6 out of 13 neurons. However, DAMGO had a dose-dependent effect on postsynaptic membrane resistance with an IC50 of 110 nM (Fig. 2D, 95% CIs: 4 nM to 9 µM). The membrane resistance was maximally reduced to 58% of the baseline value (upper 95% CI: 68%) at 3 µM DAMGO.

DAMGO also inhibited the release probability of mEPSCs (Fig. 2E & F) with an IC50 of 28 nM (95% CI: 8 to 92 nM, 9 out of 19 neurons responding).

Thus, like GABA_B_ receptors, MOP receptor activation has both pre- and postsynaptic effects. However, postsynaptic MOP receptors have a higher IC50 for DAMGO relative to presynaptic, and inhibitory inputs tend to be suppressed at lower concentrations relative to excitatory synapses.

Kappa opioid peptide receptors (KOP) are frequently associated with the aversive aspects of opioid signaling, despite their analgesic capacity (Cahill et al., 2014). Thus, their expression in PB may contribute to the negative aspects of nociception and to chronic pain conditions in particular. In contrast to the relatively consistent effects observed with GABA_B_ and MOP activation, the selective agonist, U-69593, differentially affected synaptic release probability at inhibitory and excitatory synapses.

**Fig. 3:**
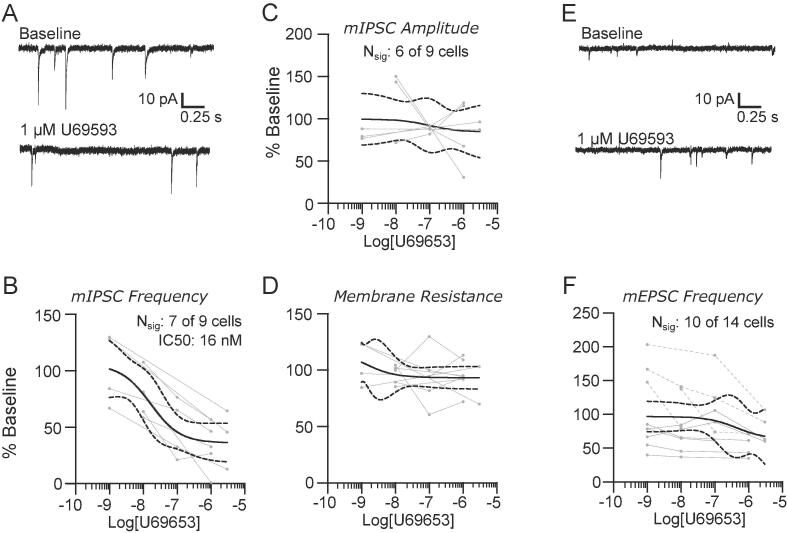
KOP receptors suppress inhibitory, but not excitatory, network activity in the lateral parabrachial nucleus. (A) Representative recordings of mIPSCs in baseline conditions (top) and in the presence of 1 µM U69593. The suppression of mIPSC frequency was dose dependent (B), but the amplitudes (C) were not affected. There was also no effect of KOP receptor activation on neuronal membrane resistance (D). Representative recordings of mEPSCs (E) and group data (F) show no consistent effect of U69593 on the frequency of excitatory events. Group data are fit with a log(dose) vs response curve ± 95% CIs.

As observed with baclofen and DAMGO, U-69593 also inhibited the frequencies of mIPSCs. Representative recordings in Fig. 3A demonstrate the suppression of mIPSC frequency following application of U-69593. Analysis of group data (Fig. 3B) revealed a dose dependent effect, with an IC50 of 16 nM (95% CIs: 1 to 200 nM, 7 out of 9 neurons responding). There was no consistent effect of U-69593 on either mIPSC amplitude (Fig. 3C) or membrane resistance (Fig. 3D).

In contrast, KOP activation had highly variable effects on mEPSC frequencies. We observed a reduction in mEPSC frequency in 6 out of 14 neurons, no significant change in 4 neurons, and the remaining 4 neurons showed a significant increase in frequency in the presence of U-69593. Sample recordings for a neuron where mEPSC frequency increased in response to this agonist are shown in Fig. 3E. Group data are shown in Fig. 3F, where neurons with a significant decrease in mEPSC frequency are depicted with solid lines and those with a significant increase are indicated with dashed lines. Both types of responses lacked a clear dose-dependent relationship, and, as a population, did not result in a meaningful IC50 value.

### 2.3 Endocannabinoids modulate excitatory and inhibitory synaptic release

**Fig. 4:**
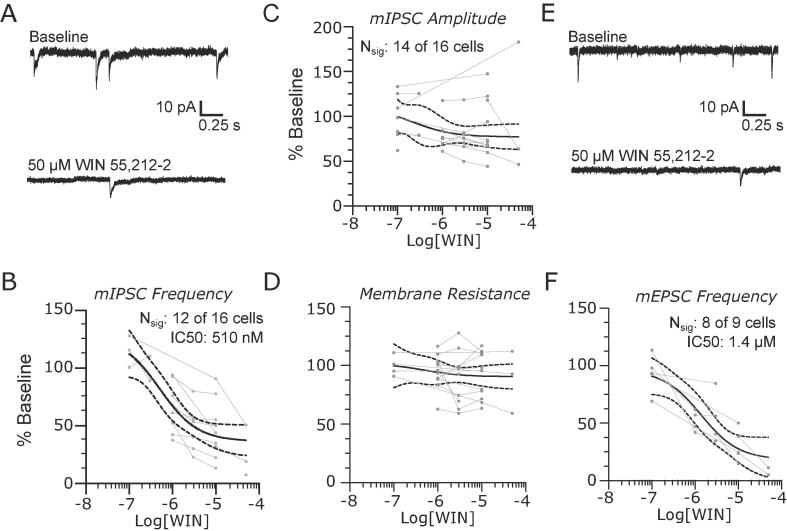
Endocannabinoid receptors modulate synaptic activity in the lateral parabrachial nucleus. (A) Representative recordings of mIPSC at baseline (top) and in the presence of 50 μM WIN 55,212–2. The CB1R agonist caused a significant dose dependent decrease in mIPSC frequency (C) without consistently affecting the event amplitudes (D) or postsynaptic membrane resistance (D) Representative recordings of mEPSC at baseline and in the presence of 50 µM WIN 55,212–2 show a reduction in excitatory event frequency (E) with a dose-dependence (F) Group data are fit with a log(dose) vs response curve ± 95% CIs.

Cannabinoid type 1 (CB1) receptors are widely expressed in the brain, including nuclei involved in nociception such as PB (Herkenhamet al., 1991). We investigated the impact of cannabinoid signaling by recording pharmacologically isolated mIPSCs and mEPSCs before and after bath applying increasing concentrations of WIN 55,212–2 (WIN: 0.1 to 50 µM), a CB1 receptor agonist. As observed in the representative recordings of mIPSCs in Fig. 4A, 50 µM WIN decreased the frequency of synaptic events. Dose-response analysis for the 12 of 16 neurons affected yielded an IC50 of 510 nM (Fig. 4B, 95% CI: 85 nM to 1.9 µM). As a population, median mIPSC amplitudes were not consistently altered by WIN (Fig. 4C), and we did not observe a consistent change in membrane resistance (Fig. 4D).

WIN also inhibited the frequency of mEPSCs in 8 out of 9 PB neurons. Representative traces are shown in Fig. 4E. Analysis of normalized mEPSC frequency group data for these 8 neurons yielded an IC50 of 1.4 µM (Fig. 4F, 95% CI: 0.1 to 10 µM).

Together, these data implicate CB1 receptors in regulation of synaptic release, without a direct postsynaptic effect.

### 2.4 Endocannabinoid receptors are tonically active in the lateral parabrachial nucleus

**Fig. 5:**
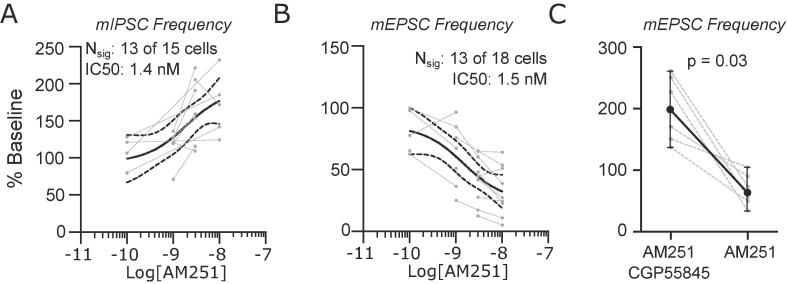
Endocannabinoid receptors are tonically active in the lateral parabrachial nucleus. (A) AM251 caused a significant dose dependent increase in mIPSC frequency but had the opposite effect on mEPSC frequency (B). This effect on mEPSC frequency was reversed by the co-application 1 µM CGP55845, a GABA_B_ receptor antagonist (C). Dose response data are fit with a log(dose) vs response curve ± 95% CIs.

Tonic endocannabinoid activity is frequently observed at CB1 receptors, and changes in the level of this tonic activity have been reported in models of chronic pain (Dogrul et al., 2002). Using the specific CB1 receptor inverse agonist AM251, we tested whether similar tonic activity is present in PB. At inhibitory synapses, applying increasing concentrations of AM251 (0.1 to 10 nM) led to a dose-dependent increase in events in 13 out of 15 neurons (Fig. 5A) with an EC50 of 1.4 nM (95% CIs: 0.04 to 70 nM).

In contrast to the effect on mIPSCs, and surprisingly, AM251 led to a dose-dependent *decrease* of mEPSC frequencies, with an IC50 of 1.5 nM (95% CIs: 0.1 to 18 nM, Fig. 5B). Because this reduction occurs during an AM251-driven increase in mIPSC release, we hypothesized that activation of GABA_B_ receptors on glutamatergic synapses may drive this counterintuitive result. We tested this hypothesis by blocking GABA_B_ receptors with 1 µM CGP55845, a selective GABA_B_ receptor antagonist, before applying 3 nM AM251. This resulted in an increase in mEPSC frequency, similar to that observed for GABAergic synapses (Fig. 5C). The net inhibitory effect of AM251 on mEPSC frequency was restored after washout of CGP55845 (Paired Wilcoxon test, p = 0.03, n = 6 neurons). This finding suggests that the efficacy of tonic CB1 signaling at glutamatergic synapses in PB is regulated by GABA_B_ receptors.

## 3 Discussion

We tested the hypothesis that GABA_B_, µ- and κ-opioid and CB_1_ receptors modulate synaptic activity in the lateral parabrachial nucleus (PB), a brain region involved in monitoring diverse homeostatic functions, including nociception. We report that graded activation of all four pathways modulate synaptic release at GABAergic and glutamatergic synapses, with greater consistency and lower IC50 values for inhibitory inputs. Furthermore, we find that CB1R are constitutively or tonically activated at both synapses, but that the impact of CB1R signaling at excitatory synapses is regulated by GABA_B_ receptors. These differences offer insights into the functional significance these pathways may have in the regulation of PB excitability.

### 3.1 GABA_B_ receptors diminish pre- and postsynaptic excitability

We show that the GABA_B_ receptors regulate presynaptic release of both glutamate and GABA from synapses with PB neurons. The presynaptic receptors at both types of inputs had similar affinities to baclofen. Because synaptically released GABA typically has to diffuse for longer distances to affect glutamatergic synapses—compared to the shorter distances to affect GABA_B_ auto-receptors—it is likely that GABA has a more potent effect on inhibitory than on excitatory synapses in PB. In line with prior reports (Christie and North, 1988) we demonstrate that activation of these receptors also reduced the input resistance of PB neurons, consistent with a postsynaptic effect. Thus, GABA_B_ mediated signaling likely suppresses transmission in PB under normal conditions.

We have recently shown that PB receives dense GABAergic innervation from the central nucleus of the amygdala (CeLC, the “nociceptive amygdala”), and that, in an animal model of chronic pain, this inhibitory pathway is suppressed (Raver et al., 2020). We also demonstrated that this suppression is causally related to chronic pain (Raver et al., 2020). The suppressed release of GABA may lead to the amplified activity of PB neurons, seen in chronic pain conditions (Uddin et al., 2018, Raver et al., 2020) through at least two mechanisms. Reduced GABA release may lead to reduced activation of postsynaptic GABA_B_ receptors, resulting in dis-inhibition of PB neurons and amplification of responses to nociceptive inputs (Uddin et al., 2018, Raver et al., 2020).

Reduced activation of GABA_B_ may also lead to more profound postsynaptic changes. For example, in the spinal cord, GABA_B_ receptors are essential for modulating after-discharges (Russo et al., 1998). After-discharges may be causally related to the expression of chronic pain (Laird and Bennett, 1993, Asada et al., 1996). The duration of these neuronal responses, which outlast a sensory stimulus (Woolf and King, 1987, Herrero et al., 2000), and the proportion of neurons that express them, is dramatically increased in chronic pain (Palecek et al., 1992, Laird and Bennett, 1993). We previously reported that, in PB of both rats and mice with chronic pain, the incidence and duration of after-discharges is markedly increased (Uddin et al., 2018, Raver et al., 2020), and that suppressing after-discharges significantly lessens hyperalgesia in experimental animals (Okubo et al., 2013). Our current findings are consistent with regulation of PB excitability by GABA_B_ receptors, but the degree to which they contribute to nociception remains to be determined.

### 3.2 MOP and KOP have mixed effects on PB excitability

The high expression levels of µ-opioid peptide (MOP) receptors in PB (Mansour et al., 1994), including in neurons that project to the amygdala (Chamberlin et al., 1999), suggests that these receptors are key modulators of neuronal activity in PB. We find that the selective MOP agonist, DAMGO, suppresses presynaptic release of both GABA and glutamate in a dose dependent manner, indicating a reduced probability of release at these synapses. DAMGO affected inhibitory synapses at lower concentrations compared to excitatory ones (IC50s: 9 vs 50 nM), suggesting that low levels of agonist activity may facilitate transmission in PB. We also find that DAMGO activates a postsynaptic conductance with an IC50 of 110 nM, similar to values reported for DAGOL (Christie and North, 1988). Although not directly tested here, this conductance was determined to result in an inwardly rectifying potassium current (Christie and North, 1988). Together, these results suggest that, as MOP receptor activity increases, presynaptic effects precede postsynaptic ones, and that low concentrations of agonist may increase transmission by preferentially suppressing inhibitory transmission.

KOP agonists are widely expressed throughout the brain, including PB (Mansour et al., 1994), where they play a critical role in mediating the aversive aspects of nociception (Chiang et al., 2020). We find that KOP agonists preferentially suppress synaptic release at GABAergic synapses and leave glutamatergic signaling relatively intact. Consistent with prior reports (Christie and North, 1988), we did not observe a consistent direct effect of KOP on the membrane resistance of PB neurons. Thus, KOP activity in PB appears to enhance excitability within this nucleus. Although the agonists used in this study are highly specific for their respective targets, combining similar recordings with specific antagonists may further refine the contributions these receptors make in modulating excitability.

### 3.3 Cannabinoid signaling selectively regulates presynaptic activity in PB

Endocannabinoid signaling in the brain, including PB, is primarily mediated by the widely expressed cannabinoid type 1 receptors (CB1) (Herkenham et al., 1991). Activation of this pathway is antinociceptive, consistent with their ability to suppress presynaptic activity via retrograde signaling from the postsynaptic neuron (Alger, 2002, Manzanares et al., 2006, Woodhams et al., 2017, Vuckovic et al., 2018). In PB we find that WIN 55,212–2, a non-specific cannabinoid agonist, has no significant impact on the intrinsic excitability of PB neurons but reduces the probability of release at both glutamatergic and GABAergic synapses. The lower IC50 observed with inhibitory inputs suggests that CB1R expression may be higher at these synapses, as reported in other brain regions (Kano et al., 2009).

We find that CB1R in PB are either constitutively or tonically active, as application of the CB1R inverse agonist AM251 produced a dose-dependent increase in synaptic release at GABAergic synapses. In contrast, AM251 had the opposite effect on excitatory synapses and reduced the frequency of mEPSCs, an effect that was reversed by blocking GABA_B_ receptors. Thus, CB1 receptors in PB have basal activity at inhibitory and excitatory synapses, but the net impact on the latter is modulated by GABA. This suggests that, under normal circumstances, GABA_B_ signaling prevents shifts in tonic CB1R activity from increasing excitation in PB. Future investigations will examine if the reduction in GABAergic signaling observed in chronic pain removes this brake and allows shifts tonic CB1 activity to increase excitatory transmission in a pronociceptive manner. It will also be important to examine potential contributions of CB2 receptors which, although expressed less broadly and at much lower levels in the brain, appear to contribute to pathological conditions. (Lu and Mackie, 2016, Cohen et al., 2019)

Together, our results provide important insights into neuromodulatory control of synaptic transmission and excitability within PB and provide a foundation for future studies on how changes in these pathways may contribute to chronic pain.

## 4 Materials and methods

### 4.1 Animals

All animal procedures were reviewed and approved by the University of Maryland Institutional Animal Care and Use Committee and adhered to the National Institutes of Health guide for the care and use of laboratory animals and ARRIVE guidelines. We used male and female adult (~7 to 13 weeks) C57Bl6/J (n = 64, Jackson Laboratory) mice from our in-house colony. For experiments testing the effects of DAMGO we used C57Bl6/J TRPV1-ChR2 (n = 17) generated by crossing Ai32(ChR2/EYFP) with TRPV1^Cre^ mice. These mice were generated as part of an independent study. Because the frequency of synaptic events in these animals was indistinguishable from that in C57Bl6/J mice (p ≥ 0.34, Mann-Whitney U) we combined data from these strains. Similarly, because the frequency of synaptic events in males and females was indistinguishable (p = 0.68, Mann-Whitney U) we combined data from both sexes.

### 4.2 Slice preparation

Animals were deeply anesthetized with ketamine (180 mg/kg) and xylazine (20 mg/kg), and the brains were rapidly removed following decapitation. Sagittal slices through PB, 300 µm thick, were cut in ice-cold cutting artificial cerebral spinal fluid (ACSF) using a Leica VT1200s vibratome (Leica Biosystems, Buffalo Grove, IL) and transferred to warm (32–34 °C) recovery ACSF for 10–15 min. The slices were then transferred to normal ACSF at room temperature for at least 45 min before starting experiments. All solutions were continuously bubbled with a mixture of 95% oxygen and 5% CO_2_.

### 4.3 Solutions and drugs

ACSF compositions were based on the methods of Ting et al (Ting et al., 2014) and consisted of (in mM); cutting ACSF: 92 NMDG, 30 NaHCO_3_, 20 HEPES, 25 glucose, 5 Na-ascorbate, 2 thiourea, 1.25 NaH_2_PO_4_, 2.5 KCl, 3 Na-pyruvate, 0.5 CaCl_2_ and 10 MgSO_4_; normal ACSF: 119 NaCl, 2.5 KCl, 1.25 NaH_2_PO_4_, 24 NaHCO_3_, 12.5 glucose, 2 CaCl_2_ and 2 MgSO_4_. The pH and osmolarities of each were adjusted to 7.35–7.45 and 300–310 mOsm, respectively. Solutions were continuously saturated with carbogen (95% O_2_, 5% CO_2_) throughout use. For experiments targeting excitatory synaptic currents we used a pipette solution consisting of (in mM): 130 Cs-Methanesulfonate, 10 HEPES, 0.5 EGTA, 1 MgCl_2_, 2.5 Mg-ATP and 0.2 GTP-Tris. For targeting inhibitory and postsynaptic currents, we used a pipette solution consisting of (in mM): 70 K-Gluconate, 60 KCl, 10 HEPES, 1 MgCl_2_, 0.5 EGTA, 2.5 Mg-ATP, 0.2 GTP-Tris. Both pipette solutions were adjusted to a pH of 7.3 and 285 mOsm. Information on receptor agonists and antagonists are provided in Table 1.

**Table 1:** Receptor agonists and antagonists used for dose-response analysis.

Drug	Supplier	Catalog Number	Concentration
Tetrodotoxin citrate	abcam	ab120055	0.5 to 1 μM
CNQX (6-cyano-7-nitroquinoxaline-2,3-dione)	Sigma-Aldrich	C239	20 μM
APV (DL-2-Amino-5-phosphonopentanoic acid)	Sigma-Aldrich	A5282	50 μM
Gabazine	Sigma-Aldrich	S106	10 μM
Baclofen	Tocris Bioscience	0417	0.1 – 300 μM
DAMGO	abcam	ab120674	1 nM – 3 µΜ
U-69593	abcam	ab141703	1 nM – 3 μΜ
WIN-55,212–2	Axxora	BML-CR105	0.1 – 50 μM
AM251	Sigma-Aldrich	A6226	0.1 – 10 nM
CGP 55845	Tocris Bioscience	1248	1 µM

### 4.4 Electrophysiology

Whole cell patch-clamp recordings were obtained from neurons in lateral PB with a Multiclamp 700B amplifier (Molecular Devices) low-pass filtered at 1.8 kHz with a four-pole Bessel filter, and digitized with Digidata 1550B (Molecular Devices). Lateral PB is easily identified in these slices by its proximity to the superior cerebral peduncle. Recording locations were identified visually at low magnification before and after each recording for all neurons and verified with biocytin immunohistochemistry. The impedance of patch electrodes was 4–8 MΩ. Once a GΩ seal was obtained, holding potential was set to −65 mV and was maintained for the duration of the experiment. All recordings were obtained at room temperature.

Miniature inhibitory presynaptic currents (mIPSCs) were recorded in the presence of 0.5 to 1 µM TTX and 10.0 µM gabazine, while miniature excitatory presynaptic currents (mEPSCs) were recorded in the presence of 0.5 to 1 µM TTX, 20 µM CNQX and 50 µM APV, respectively. We generated dose–response profiles by serial bath application of the respective agonist or antagonist, with a minimum 3 min wash-in time per concentration, and collected only a single neuron per slice. All drugs and respective concentrations are provided in Table 1. Series resistance was monitored throughout the recordings with −5 mV hyperpolarizing pulses and we discarded recordings in which the resistance changed by more than 20% within a recording.

### 4.5 Biocytin immunohistochemistry

Upon completion of recording, the pipette was carefully retracted from the neuron and the tissue slice transferred to 10% formalin at 4 °C overnight. Slices were then washed 3 times for 10 min at room temperature in 1X PBS before being incubated overnight in a solution of 1:1000 Strepavidin – Cy3 conjugate, 3% fetal bovine serum and 0.3% Triton X-100 at 4 °C. Slices were cover-slipped with an aqueous mounting media and visualized on a Leica SP8 confocal microscope to verify the recoding location within the lateral PB. We rejected data from 2 neurons which were determined to be outside this nucleus.

### 4.6 Data analysis and statistics

Miniature inhibitory and excitatory postsynaptic currents were isolated offline using miniAnalysis (Synaptosoft). We used Clampfit (Molecular Devices) or the Neuromatic XOP for Igor (Wavemetrics) developed by Jason Rothman (Rothman and Silver, 2018) to calculate the membrane resistance based on the steady state current evoked by a −5 mV hyperpolarizing step. As cells within a given nucleus do not necessarily express receptors for all of the agonists in this study (Christie and North, 1988, Margolis et al., 2003), we separated our data into cells which did or did not respond to a particular agonist. This separation provides a better estimate of the IC50s by removing nonresponding cells that would flatten the dose response curve and artificially inflate the final IC50 value. Thus, for each neuron, we ran a Kruskal-Wallis test to determine if the median frequency of synaptic events at baseline was significantly altered (p < 0.05) by application of the agonist. To account for repeated measures within a neuron, we performed Dunn’s multiple comparison post-hoc analysis to determine if data from any individual concentration of agonist was significantly different than in its baseline recording. If there was a significant difference for at least one concentration, we included the data from that neuron in the next step, determination of the IC50. For this parameter, we fit the median frequencies or amplitudes from all of the neurons that showed a significant response to an agonist with a three parameter [inhibitor] vs. response model in GraphPad Prism. If the fit was successful, we report the IC50 with 95% confidence intervals. We report the total number of neurons recorded and the fraction of neruons that showed a significant response to the agonist, as defined above. mIPSC/mEPSC frequency and amplitude were analyzed independently.

## Funding

This work was supported by National Institutes of Health National Institute of Neurological Disorders and Stroke Grants R01NS099245 and R01NS069568. The content is solely the responsibility of the authors and does not necessarily represent the official views of the National Institutes of Health. The funding sources had no role in study design; the collection, analysis and interpretation of data; the writing of the report; or in the decision to submit the article for publication.

Support was provided also by 2019/12439-3, São Paulo Research Foundation (FAPESP) to Gleice Cardoso.

## CRediT authorship contribution statement

**Nathan Cramer:** Conceptualization, Investigation, Formal analysis. **Gleice Silva-Cardoso:** Conceptualization, Investigation, Formal analysis, Writing - original draft, Funding acquisition. **Radi Masri:** Conceptualization, Formal analysis, Writing - review & editing. **Asaf Keller:** Conceptualization, Investigation, Writing - review & editing, Funding acquisition, Resources.

## Declaration of Competing Interest

The authors declare that they have no known competing financial interests or personal relationships that could have appeared to influence the work reported in this paper.
